# Toward the Improvement of Maleic Anhydride Functionalization in Polyhydroxybutyrate (PHB): Effect of Styrene Monomer and Sn(Oct)_2_ Catalyst

**DOI:** 10.3390/ijms241914409

**Published:** 2023-09-22

**Authors:** Matheus Ferreira de Souza, Carlos Bruno Barreto Luna, Danilo Diniz Siqueira, Ewerton de Oliveira Teotônio Bezerra, Grazielle Rozendo de Cerqueira, Edcleide Maria Araújo, Renate Maria Ramos Wellen

**Affiliations:** 1 Academic Unit of Materials Engineering, Federal University of Campina Grande, Av. Aprígio Veloso, 882-Bodocongó, Campina Grande 58429-900, PB, Brazil; matheusferza@gmail.com (M.F.d.S.); danilodinizsiqueira@gmail.com (D.D.S.); edcleidemaraujo@gmail.com (E.M.A.); 2 Department of Materials Engineering, Federal University of São Carlos, Jardim Guanabara, São Carlos 13565-905, SP, Brazil; ewerton.teotonio@hotmail.com; 3 Department of Materials Science, Federal University of Pernambuco, Av. da Arquitetura-Cidade Universitária, Recife 50740-540, PE, Brazil; grazielle.rozendo@ufpe.br; 4 Department of Materials Engineering, Federal University of Paraíba, Cidade Universitária, João Pessoa 58051-900, PB, Brazil

**Keywords:** polyhydroxybutyrate, chemical modification, maleic anhydride, Tin(II) 2-ethylhexanoate, styrene monomer

## Abstract

In this work, polyhydroxybutyrate (PHB) was maleic anhydride (MA)-grafted in the molten state, using dicumyl peroxide (DCP) as a reaction initiator. Tin(II) 2-ethylhexanoate (Sn(Oct)_2_) and styrene monomer (St.) were used to maximize the maleic anhydride grafting degree. When PHB was modified with MA/DCP and MA/DCP/Sn(Oct)_2_, viscosity was reduced, suggesting chain scission in relation to pure PHB. However, when the styrene monomer was added, the viscosity increased due to multiple grafts of MA and styrene into the PHB chain. In addition, the FTIR showed the formation of a new band at 1780 cm^−1^ and 704 cm^−1^, suggesting a multiphase copolymer PHB-g-(St-co-MA). The PHB (MA/DCP) system showed a grafting degree of 0.23%; however, the value increased to 0.39% with incorporating Sn(Oct)_2_. The highest grafting efficiency was for the PHB (MA/DCP/St.) system with a value of 0.91%, while the PHB (MA/DCP/St./Sn(Oct)_2_) hybrid mixture was reduced to 0.73%. The chemical modification process of PHB with maleic anhydride increased the thermal stability by about 20 °C compared with pure PHB. The incorporation of 0.5 phr of the Sn(Oct)_2_ catalyst increased the efficiency of the grafting degree in the PHB. However, the St./Sn(Oct)_2_ hybrid mixture caused a deleterious effect on the maleic anhydride grafting degree.

## 1. Introduction

Synthetic polymers derived from petroleum have become precious materials for modern life, with many applications [1]. The success of commodity polymers such as polypropylene (PP), polyethylene (PE), and polystyrene (PS) is mainly attributed to their incredible versatility, durability, corrosion resistance, and intrinsic properties [2,3]. However, the improper disposal of these materials in nature has caused severe environmental problems, as they have accumulated in the environment for decades [4,5]. In particular, commodity polymers resist biological attack, generating excellent environmental durability. Consequently, decomposing can take more than a hundred years, causing environmental and social problems, especially in large urban centers. Given this, an expansion in the development of ecological polymers has been necessary [6,7,8,9]. As a result of the growing environmental awareness of the population and the increased demand for sustainable products [10,11]. In this context, biodegradable polymers have gained notoriety for promoting the use of ecologically correct raw materials, contributing to sustainability, and reducing the life cycle in the environment [12,13]. Poly(lactic acid) (PLA), poly(ε-caprolactone), and polyhydroxybutyrate (PHB) are biodegradable polymers with broad technological potential for the plastics transformation sector, aiming at the production of food packaging, bags, and products for agriculture [14,15,16]. In addition, they minimize the environmental impact and pollution on natural ecosystems and, therefore, fit the concept of sustainability [17,18].

PHB is a biodegradable and renewable thermoplastic polyester synthesized by microorganisms from the fermentation of sugars or other carbon sources [19]. It is a hard and brittle material, flows easily during processing, is not very permeable to O_2_, H_2_O, and CO_2_, and is not soluble in water [20]. The PHB glass transition temperature (T_g_) is between −5 and 5 °C, and the melting temperature (Tm) is 175 °C [21,22]. The mechanical properties of PHB are similar to those of polystyrene, which generates the potential for producing polymeric blends, composites, and ecological nanocomposites. However, to produce polymer blends and composites of PHB with other biodegradable polymers, it is necessary to modify the PHB to increase the polarity of the chain. This makes it possible to increase the interfacial interaction between mixtures of biodegradable polymers and natural fiber composites, which will undoubtedly improve the mechanical properties. In view of this, the new functional group can increase the polarity of PHB, contributing to interactions with hydroxyl and carbonyl groups present in natural fibers and polyester-based biopolymers. Incorporating new functional groups into the PHB chain is an alternative, generating an expanded range of applications. The grafting of PHB with maleic anhydride (PHB-g-MA) is important for the plastics processing sector, with a view to forming a functionalized biodegradable polymer for application as a compatibilizer. The synthesis of PHB-g-MA can produce materials with characteristics that contribute to an environmentally sustainable cycle, especially composites with natural fibers, blends with biodegradable polymers, and biopolymer-based nanocomposites. The chemical modification of biodegradable polymers PLA and PCL with maleic anhydride (MA) has been explored in the literature [23,24,25,26,27], enabling new reactive materials to produce blends and composites.

PHB has also been explored in the process of chemical modification with maleic anhydride, with a view of technological applications. In work [28], 0.54% was reached as the maximum degree of maleic anhydride grafting in the PHB chain, suggesting a low efficiency in the chemical modification process under cobalt-60 radiation. Hong et al. [29] functionalized PHB with maleic anhydride (MA) and benzoyl peroxide (BPO) using an MA/BPO ratio of 5/1 phr (parts per hundred of resin). The authors verified that the PHB grafted in solution has an MA engraftment rate of around 0.07%, while the PHB grafted by fusion is about 1.54%. Furthermore, the 0.7% MA graft significantly improved the PHB thermal stability and crystallization. Chen et al. [30] synthesized PHB grafted with maleic anhydride (MA) by polymerization, using benzoyl peroxide (BPO) as an initiator. MA and BPO concentrations played an essential role in the chemical modification of PHB since the degree of engraftment could be controlled in the range of 0.2 to 0.85%. The authors verified a maximum grafting peak of 0.85% when the concentration of MA was 3% and 0.2% of BPO at a temperature of 130 °C. In addition, the thermal stability of PHB-g-MA has been improved by about 20 °C compared with pure PHB.

The efficiency of grafting maleic anhydride into the PHB chain is low. Consequently, additives are applied during processing to maximize the grafting of functional groups into the polymeric chain [31,32]. Hu et al. [33] observed that using styrene (St) as a comonomer significantly reduced the occurrence of secondary reactions and increased the degree of grafting of the MA in the PP. The styrene monomer can react with the macroradicals of the polymers more quickly than the functionalizing monomers (maleic anhydride). As a result, the conversion of reactive macroradicals into styrene-stabilized macroradicals. The stabilized macroradical can react with the functionalizing monomer more efficiently [34,35]. Given the potential of styrene monomer to improve the degree of grafting in the polymer chain, an alternative is observed to enhance the functionalization of PHB with maleic anhydride. The scientific literature is scarce on the chemical modification of PHB with maleic anhydride and styrene monomer (St.), which constitutes a relevant point to be investigated. In addition, no mention was found in the literature dealing with the combined effect of the styrene monomer with the Tin(II) 2-ethylhexanoate catalyst, aiming at maleic anhydride grafting into the PHB chain. This proposal is a justification for the present investigation, given that it can enhance the efficiency of PHB functionalization, which is important for the industrial and scientific sectors.

The present investigation aimed to functionalize PHB with maleic anhydride in the molten state, using dicumyl peroxide (DCP) as the initiator. At the same time, styrene monomer and Tin(II) 2-ethylhexanoate catalysts will be used as auxiliary additives to enhance the efficiency of MA grafting.

## 2. Results and Discussion

### 2.1. Torque Rheometry

**Figure 1:**
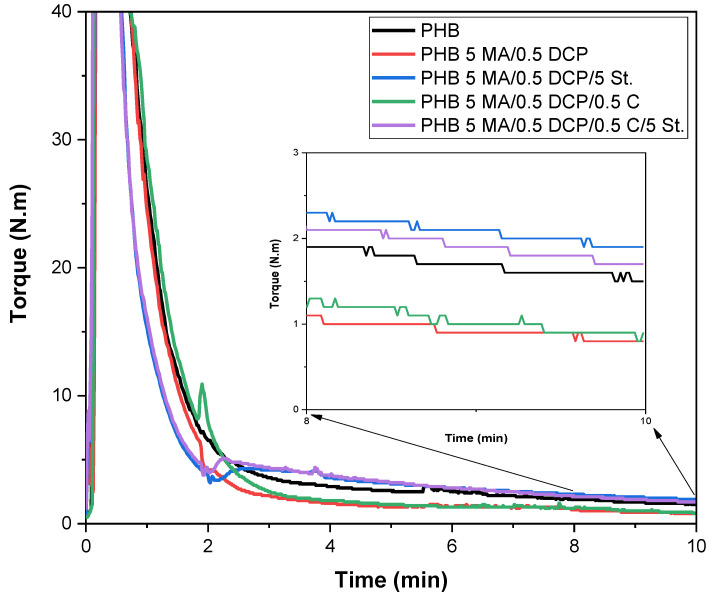
Torque rheometry curves of pure PHB and PHB-g-MA.

Torque rheometry analysis is essential for assessing the impact of additive incorporation during PHB processing in order to verify the evidence of degradation, crosslinking, or chemical reactions. The crosslinking process in the PHB chain is not desirable during the MA grafting process. This promotes a competitive effect between crosslinking and maleic anhydride functionalization, thus reducing the degree of MA grafting. Therefore, the test by torque rheometry is important for controlling the DCP content and, consequently, minimize possible crosslinking. Figure 1 shows the torque rheometry curves of pure PHB and PHB-g-MA as a function of chemical modification additives. Table 1 presents the average stabilized torque and the viscosity of the PHB before and after the chemical modification process with maleic anhydride. The literature [36,37] reports that the torque is directly proportional to the viscosity of the polymeric system. Given this, an increase in torque is taken as an indication that the viscosity has increased. 

In Figure 1, pure PHB showed a higher torque rheometry curve than PHB-g-MA (5 MA/0.5 DCP) and PHB-g-MA (5 MA/0.5 DCP/0.5 C) systems. The stabilized torque of pure PHB was in the order of 2.1 N.m, while PHB-g-MA (5 MA/0.5 DCP) and PHB-g-MA (5 MA/0.5 DCP/0.5 C) reduced by 42.8% and 38.1%, respectively. This behavior suggests that dicumyl peroxide (DCP) caused chain scission in PHB, considering that DCP was an initial source of free radicals. The generation of free radicals occurs through the thermal decomposition of the peroxide bond (O-O) present in DCP, forming a macroradical and initiating the process of hydrogen abstraction from PHB. Consequently, the viscosity of the PHB-g-MA (5 MA/0.5 DCP) and PHB-g-MA (5 MA/0.5 DCP/0.5 C) systems followed a downward trend, with the lowest values of 22.3 Pa.s and 25.1 Pa.s, respectively. The PHB-g-MA (5 MA/0.5 DCP/0.5 C) system has an average torque of 1.3 N.m, slightly higher than the PHB-g-MA (5 MA/0.5 DCP) system with 1.2 N.m. Although there is a low difference in mean torque between the PHB-g-MA (5 MA/0.5 DCP/0.5 C) and PHB-g-MA (5 MA/0.5 DCP) compounds, there was a change in viscosity. As shown in Table 1, the PHB-g-MA (5 MA/0.5 DCP/0.5 C) system had a 12.5% increase in viscosity, compared with PHB-g-MA (5 MA/0.5 DCP). At the end of processing in 10 min, the Sn(Oct)_2_ catalyst minimizes the chain scission process in the PHB-g-MA (5 MA/0.5 DCP) system, reflecting on the increase in viscosity and maleic anhydride grafting, as verified later in FTIR. This behavior is attributed to the ability of the Sn(Oct)_2_ catalyst to act on the DCP, promoting a reduction in the number of macroradicals formed during the grafting process, as demonstrated in [38]. In addition, the smaller amount of macroradicals minimizes the attack on the PHB chain, reducing the level of chain scission and generating higher viscosity, as verified in the PHB-g-MA (5 MA/0.5 DCP/0.5 C) system.

During melt processing, the DCP-based initiator will be decomposed into two primary free radicals [39]. Primary radicals may initiate functional monomers, initiate macroradicals, or react with each other (recombination). However, the Sn(Oct)_2_ catalyst can act on the DCP molecule, promoting the formation of only one primary radical [38]. Given the above, the PHB-g-MA (5 MA/0.5 DCP) system showed a more significant amount of free radicals to attack the PHB chain more severely, generating a higher level of chain scission and lower viscosity. In contrast, adding 0.5 phr of Sn(Oct)_2_ in the PHB-g-MA (5 MA/0.5 DCP/0.5 C) system inhibited an excessive formation of free radicals. This resulted in higher viscosity, lower level of recombination, and higher effectiveness in maleic anhydride grafting, as verified further in titration and FTIR.

When the PHB-g-MA (5 MA/0.5 DCP) system was activated with styrene monomer, a change in the behavior of the torque curve was observed. The PHB-g-MA (5 MA/0.5 DCP/5 St.) sample presented an average torque of 2.6 N.m, corresponding to a gain of 23.8% over pure PHB. The sharper increase in torque of the PHB-g-MA (5 MA/0.5 DCP/5 St.) system suggests an improvement in the efficiency of the grafting reaction. This behavior was accompanied by a more significant increase in viscosity (52.9 Pa.s), considering the higher value for the PHB-g-MA (5 MA/0.5 DCP/5 St.) system. From a performance point of view, the increase in torque is possibly associated with the higher molecular weight of PHB-g-MA (5 MA/0.5 DCP/5 St.). This finding becomes important for the PHB-g-MA application as a compatibilizer for polymer blends and composites, especially for forming a strong interface and a stable morphology [40]. The PHB-g-MA (5 MA/0.5 DCP) system had low torque and lower viscosity, but the styrene monomer affected the reaction kinetics during processing. There was an extension of the PHB chain with the incorporation of styrene monomer and maleic anhydride, resulting in less fluidity and increased torque. The FTIR analysis confirmed this hypothesis with a new band at 704 cm^−1^, referring to the styrene grafting onto the chain. The aromatic group in styrene generates a more significant barrier to molecular movement and, consequently, imposes greater resistance to fluidity. Given this, it is suggested that the PHB (5 MA/0.5 DCP/5 St.) composition formed a multiphase component PHB-g-(St-co-MA). Similar behavior was seen in the literature [41,42], suggesting that the styrene monomer can increase the degree of grafting by up to 3–4× for the functional monomer (maleic anhydride and glycidyl methacrylate). However, the hybridization process between the styrene monomer (St.) and the Sn(Oct)_2_ catalyst impaired the functionalization process of the PHB (5 MA/0.5 DCP/5 St./0.5 C) system, given the reduced torque and viscosity compared with PHB (5 MA/0.5 DCP/5 St.). Therefore, two explanations for the behavior are: (a) by inducing a smaller amount of radicals in the reaction medium, as previously discussed, the Sn(Oct)_2_ catalyst limited the reactive points for maleic anhydride; (b) the Sn(Oct)_2_ catalyst minimized the effect of styrene monomer in increasing the electron density in maleic anhydride doubles. It is important to point out that the PHB (5 MA/0.5 DCP/5 St./0.5 C) system decreased the efficiency in the degree of grafting in comparison with the PHB (5 MA/0.5 DCP/5 St.). However, average torque and viscosity higher than pure PHB were still observed, maintaining the character of a PHB-g-(St-co-MA) multiphase component.

**Figure 2:**
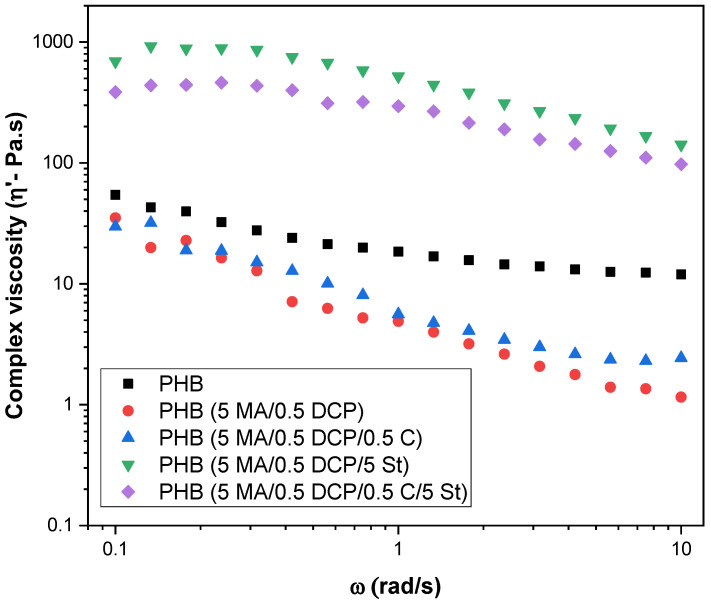
Complex viscosity of pure PHB and PHB-g-MA modified with maleic anhydride, dicumyl peroxide, styrene and Sn(Oct)_2_.

Figure 2 shows the complex viscosity (ƞ’) as a function of the angular frequency (ω) of pure PHB and PHB-g-MA. The analysis was carried out in the linear viscoelastic regime, bearing in mind that the data obtained in this regime are sensitive to small differences or changes in structure [43,44]. Given this, the modification in the molecular structure of PHB with maleic anhydride, dicumyl peroxide, styrene, and Sn(Oct)_2_ will be better understood. It was observed that pure PHB had a higher complex viscosity compared with PHB-g-MA (5 MA/0.5 DCP) and PHB-g-MA (5 MA/0.5 DCP/0.5 C) systems. As complex viscosity is directly related to molecular weight [45], chain scission was predominant in PHB-g-MA (5 MA/0.5 DCP) and PHB-g-MA (5 MA/0.5 DCP/0.5 C) samples due to the decrease in viscosity. In comparative terms, the PHB-g-MA (5 MA/0.5 DCP/0.5 C) system showed a slight increase in viscosity compared with the PHB-g-MA (5 MA/0.5 DCP) system. This behavior can be attributed to higher maleic anhydride grafting in the PHB-g-MA (5 MA/0.5 DCP/0.5 C) sample, as observed by FTIR. On the other hand, the greater fluidity of the PHB-g-MA (5 MA/0.5 DCP) sample suggests an excess of dicumyl peroxide in the reaction medium. In this case, it caused a higher chain scission and more severe viscosity reduction.

The viscosity increased significantly when styrene monomer (St.) was added, especially for PHB (5 MA/0.5 DCP/5 St.). There was an increase in molar mass, reinforcing the suggestion that the styrene monomer was incorporated into the PHB chain, generating the multiphase component PHB-g-(St-co-MA). Consequently, there is a greater restriction for molecular mobility and resistance to flow during deformation. Finally, it was observed that the Sn(Oct)_2_ catalyst mixed simultaneously with styrene monomer interfered in reducing the complex viscosity, as verified by the PHB (5 MA/0.5 DCP/5 St./0.5 C) system. Apparently, there was a deleterious effect on the grafting reaction mechanism with the simultaneous addition of St./Sn(Oct)_2_. The combination of Sn(Oct)_2_ and styrene monomer, as used in this work, generates only one free radical at DCP, thus reducing the number of reactive points to grafting the maleic anhydride onto the PHB chain. This is evidence that there was a suppression of free radicals, reducing the ability to react with maleic anhydride. On the other hand, the reduction in the degree of grafting in the PHB chain (5 MA/0.5 DCP/5 St./0.5 C) generated a lower molecular restriction for flow and reduced the complex viscosity in relation to the PHB (5 MA/0.5 DCP/5 St.) system. These complex viscosity results corroborate the behavior of torque rheometry. As shown in Table 1, the higher torque promoted an increase in viscosity, following the trend of complex viscosity. In other words, the PHB system (5 MA/0.5 DCP/5 St.), due to its higher viscosity associated with torque, also obtained the highest complex viscosity.

### 2.2. Grafting Degree of Maleic Anhydride

**Figure 3:**
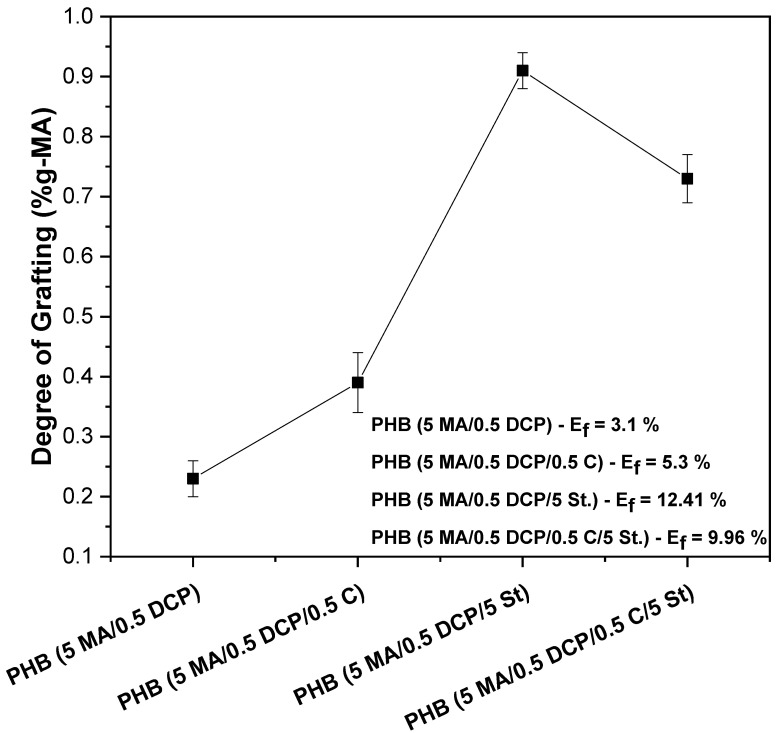
Results of the grafting degree of PHB-g-MA.

Figure 3 shows the results of the degree of grafting of PHB-g-MA as a function of styrene (St.), maleic anhydride (MA), DCP, and Sn(Oct)_2_ additives. The PHB (5 MA/0.5 DCP) system showed a low grafting degree with a value of 0.23% of maleic anhydride, indicating a low efficiency in functionalization and confirming the tendency of the literature [46]. Ma et al. [46] prepared PHB grafting with maleic anhydride in the molten state using the MA/DCP ratio (4.5/0.5 phr). The authors obtained a degree of grafting of maleic anhydride in the order of 0.25%, suggesting difficulty grafting MA into the PHB chain. When 0.5 phr of Sn(Oct)_2_ catalyst was added to the PHB (5 MA/0.5 DCP) system, the maleic anhydride grafting degree increased to 0.39%, corresponding to a 69.6% gain in functionalization. This behavior suggests that the Sn(Oct)_2_ catalyst can act directly on DCP, generating an ideal amount of free radicals to react with maleic anhydride and optimizing the grafting degree. It was observed that the degree of grafting increased significantly with the addition of styrene monomer. A very evident increase in the grafting degree was observed for the PHB (5 MA/0.5 DCP/5 St.) system, with a value of 0.91%. This corresponds to an approximately 4x increase in maleic anhydride grafting compared with initiation by thermal decomposition exclusively by DCP (PHB—5 MA/0.5 DCP). As reported in the literature [47,48], styrene monomers can donate electrons to maleic anhydride, generating a charge transfer complex (CTC). Given this, the double bond of MA significantly increases the electronic density in the St./MA complex, which causes an increase in radical reactivity and, consequently, increases the degree of grafting. The PHB (5 MA/0.5 DCP/5 St.) system indicated that the styrene monomer significantly reduced the electron deficiency in maleic anhydride unsaturation, generating the highest efficiency in the degree of grafting. Interestingly, the hybrid mixture of styrene with the Sn(Oct)_2_ catalyst caused a deleterious effect on the efficiency of the grafting degree of the PHB (5 MA/0.5 DCP/5 St./0.5 C) sample, with a value of 0.73% of grafting. This decrease was attributed to the lower amount of primary radicals in the reaction medium incorporating the Sn(Oct)_2_ catalyst, as discussed in the torque rheometry.

The PHB (5 MA/0.5 DCP/5 St.) and PHB (5 MA/0.5 DCP/5 St./0.5 C) systems showed the highest values of maleic anhydride grafting degree, which was reflected in the increase in viscosity, as verified in torque rheometry and complex viscosity. This fact confirms the incorporation of maleic anhydride and styrene monomer in the PHB chain, generating a greater flow restriction of the PHB-g-MA in the molten state. Furthermore, with the increase in the degree of MA grafting, there was also an increase in the flow resistance of PHB, which caused an increase in the viscosity value. Similar behavior was reported in the literature [39], indicating that incorporating maleic anhydride as a pendant or branched group in the polymer chain reduces the chain’s segmental mobility, increasing flow resistance (higher viscosity).

### 2.3. Fourier Transform Infrared Spectroscopy (FTIR)

**Figure 4:**
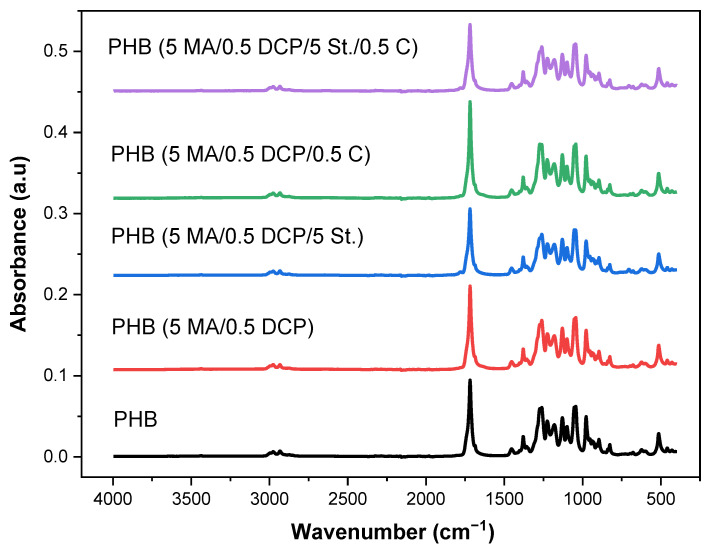
FTIR spectra of pure PHB and PHB-g-MA modified with maleic anhydride, dicumyl peroxide, styrene and Sn(Oct)_2_.

The FTIR technique is an analysis of great interest in evaluating the chemical modification of polymers, as demonstrated in the literature [49,50]. In Figure 4, the prominent PHB absorption bands are observed before and after chemical modification with styrene (St.), maleic anhydride (MA), DCP, and Sn(Oct)_2_. The bands at 2975 cm^−1^, 2932 cm^−1^, and 2834 cm^−1^ are related to the asymmetrical stretch of the methyl group (CH_3_), the anti-symmetrical stretch of the methylene group (CH_2_), and the symmetrical stretch of the methyl group (CH_3_) [51], respectively. The literature [52] indicated that the 2975 cm^−1^ band in PHB is attributed to the asymmetrical stretch mode of the CH_3_ group of the crystalline parts. A high absorption band at 1720 cm^−1^ corresponds to carbonyl (C=O), a value similar to that verified by Cuellar et al. [53]. The bands at 1453 cm^−1^ and 1380 cm^−1^ are characteristic of the asymmetrical in-plane bending vibration of the C-H bond of the CH_2_ group, and symmetrical in-plane bending vibration of the methyl group (CH_3_) [54], respectively. In addition, the absorption bands found in the region of 1000–1300 cm^−1^ correspond to the stretch vibrations of the C-O-C group: 1273 cm^−1^ (C-O-C stretch of the crystalline fraction), 1260 cm^−1^ (C-O-C stretch and deformation of the CH group of the crystalline fraction), 1224 cm^−1^ (C-O-C stretch of the amorphous fraction), 1180 cm^−1^ and 1100 cm^−1^ (C-O-C stretch), 1053 cm^−1^ (C-O group stretch) and 1043 cm^−1^ (C-CH_3_ stretch). Bands at 977 cm^−1^, 938 cm^−1^, 895 cm^−1^, and 825 cm^−1^ attributed to the C–C stretch were observed, suggesting the helical structure of the PHB chain [55]. Therefore, the bands observed in the FTIR spectrum correspond to the chemical bonds of the groups present in the PHB.

**Figure 5:**
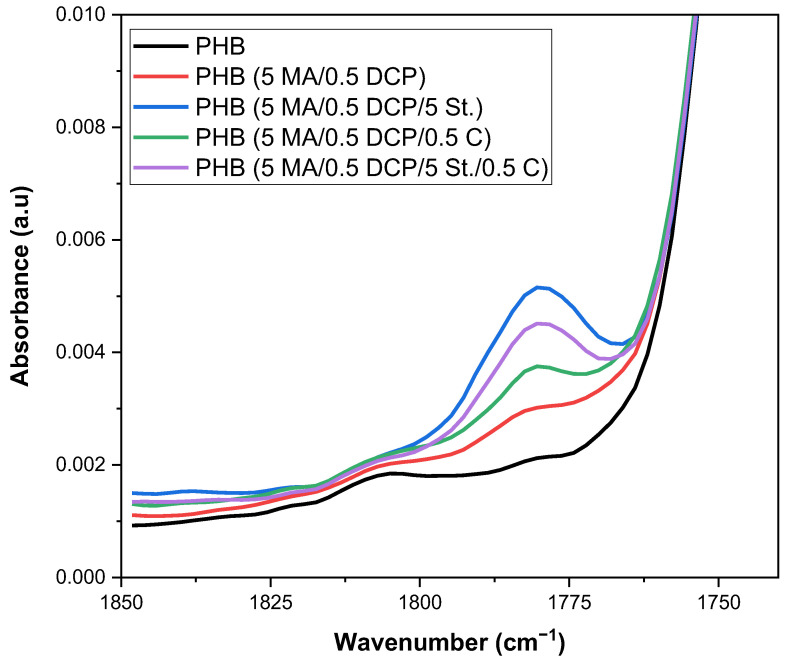
FTIR spectra of pure PHB and PHB-g-MA in the region of 1750–1850 cm^−1^.

Figure 5 shows an enlargement of the FTIR spectrum in the 1750–1850 cm^−1^ region of PHB and PHB-g-MA, aiming to evaluate the maleic anhydride band. The absorption band referring to the axial deformation of the maleic anhydride carbonyl is located in the region of 1850–1790 cm^−1^, as demonstrated in the literature [43,56]. Chemical modification of PHB with maleic anhydride was confirmed by FTIR, as shown in Figure 5. After the chemical modification process, a new band appeared at 1780 cm^−1^ in the PHB chain. This behavior indicates that carbonyl groups of maleic anhydride were grafted, generating PHB-g-MA. Due to the small amount of MA that reacted on the PHB chain, the PHB (5 MA/0.5 DCP) system showed a minimal and discrete band at 1780 cm^−1^ in the FTIR spectrum, confirming the low degree of grafting, as verified in the titration. Maleic anhydride grafting onto PHB through the molten state is difficult, leading to low functionalization efficiency.

In Figure 5, it was observed that the incorporation of the Sn(Oct)_2_ catalyst in the PHB (5 MA/0.5 DCP) system promoted an increase in the characteristic band of MA at 1780 cm^−1^, which is taken as an indication that more maleic anhydride was grafted onto the PHB chain. The PHB spectrum (5 MA/0.5 DCP/0.5 C) shows that 0.5 phr of Sn(Oct)_2_ can act as a catalyst additive to improve the grafting degree and the efficiency of the grafting reactions in the molten state. A limiting factor in grafting reactions initiated by free radicals in the molten state is the low efficiency of the initiator [57]. When thermally decomposed, the initiator will produce two primary free radicals [58]. Primary radicals may initiate monomers, initiate macroradicals, or undergo recombination (*cage effect*). The recombination process is responsible for reducing the efficiency of the primers once they react with each other. This results in a lower efficiency of the starter during the melt-grafting process. However, the addition of the Sn(Oct)_2_ catalyst has the potential to minimize the effect of initiator recombination, generating only one primary radical [43,59]. As the PHB (5 MA/0.5 DCP/0.5 C) system increased band intensity at 1780 cm^−1^ compared with PHB (5 MA/0.5 DCP), it is suggested that 0.5 phr of Sn(Oct)_2_ was able to inhibit or minimize the effect of recombination. Consequently, the grafting degree of maleic anhydride onto PHB (5 MA/0.5 DCP/0.5 C) was improved in the molten state, confirming the titration trend.

The band at 1780 cm^−1^ increased significantly for the PHB (5 MA/0.5 DCP/5 St.) system, indicating that the grafting degree of maleic anhydride was maximized in the PHB chain. This suggests inserting more lateral branches into the PHB (5 MA/0.5 DCP/5 St.) chain, restricting molecular mobility. With an increase in the degree of MA grafting, that is, an increase in the number of grafted chains, there is also an increase in flow restriction, leading to higher viscosities and confirming the rheology results. The styrene monomer (St.) was an additive capable of stabilizing the PHB (5 MA/0.5 DCP/5 St.) system during processing in the molten state, generating greater efficiency of the maleic anhydride grafting in the PHB. The literature [60] suggests that the styrene monomer can react more quickly with the macroradicals of the polymer compared with the functional monomer that will be grafted. Consequently, macroradicals are formed and stabilized by the styrene monomer, which can copolymerize with the functionalizing monomer. This finding may generate a more balanced reaction system during the grafting process, increasing the probability of a higher degree of grafting, as verified for PHB (5 MA/0.5 DCP/5 St.). Interestingly, the hybrid additive process between styrene monomers (St.):Sn(Oct)_2_ catalyst in the ratio 5:0.5 phr caused a deleterious effect on the PHB maleic anhydride grafting process, as verified in the band at 1580 cm^−1^ of the PHB (5 MA/0.5 DCP/5 St./0.5 C) system. In view of this, two justifications are suggested to explain the observed behavior: (1) there was a competitive effect between the styrene monomer (St.) and the Sn(Oct)_2_ catalyst, generating an inhibition of the MA grafting process in the PHB chain; (2) as the Sn(Oct)_2_ catalyst originates only one free radical in the DCP molecule, there was a restriction on the number of reactive molecules to react with the functional groups of maleic anhydride, generating a lower degree of grafting. For the specific formulation of PHB, the hybrid mixture between St./Sn(Oct)_2_ did not become viable, given the reduction in the efficiency of the grafting degree.

**Figure 6:**
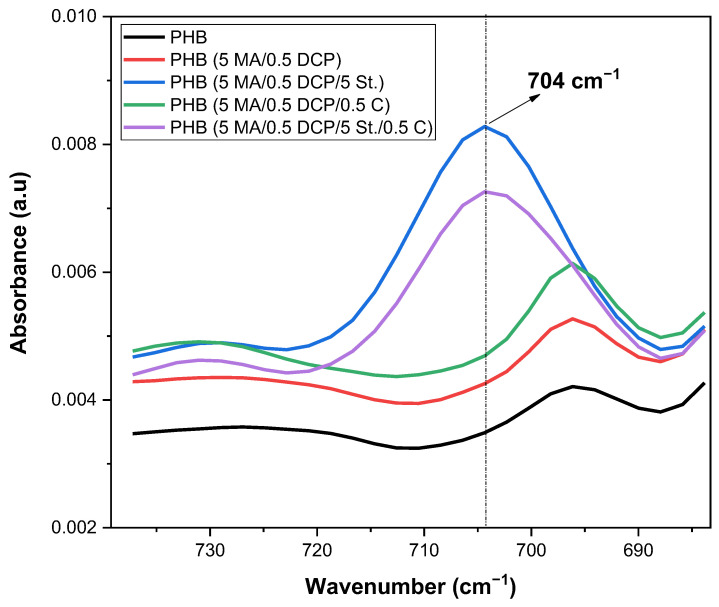
FTIR spectra of pure PHB and PHB-g-MA in the 600–750 cm^−1^ region.

Figure 6 shows the magnification region in the 600–750 cm^−1^ range of the FTIR spectrum, aiming to understand the behavior of the styrene monomer (St.) in the PHB chain. The PHB (5 MA/0.5 DCP/5 St.) and PHB (5 MA/0.5 DCP/5 St./0.5 C) systems presented a new band at 704 cm^−1^, corresponding to the out-of-plane bending vibration of the aromatic nucleus of the styrene monomer [41]. The presence of the 704 cm^−1^ band suggests that styrene was grafting onto the PHB chain, generating a multiphase component with maleic anhydride (PHB-g-(St-co-MA)). The band intensity at 704 cm^−1^ characteristic of styrene was observed to be more significant for the PHB (5 MA/0.5 DCP/5 St.) system, which means more considerable styrene grafting. This finding corroborates the trend towards higher viscosity of the PHB (5 MA/0.5 DCP/5 St.) system, as verified in torque rheometry and complex viscosity. In addition, incorporating the Sn(Oct)_2_ catalyst did not improve the grafting degree of styrene in the PHB (5 MA/0.5 DCP/5 St./0.5 C) system. This reinforces the hypothesis of the previous discussion about the competitive effect between styrene and Sn(Oct)_2_, as well as a smaller amount of reactive points to react between the molecules. 

**Figure 7:**
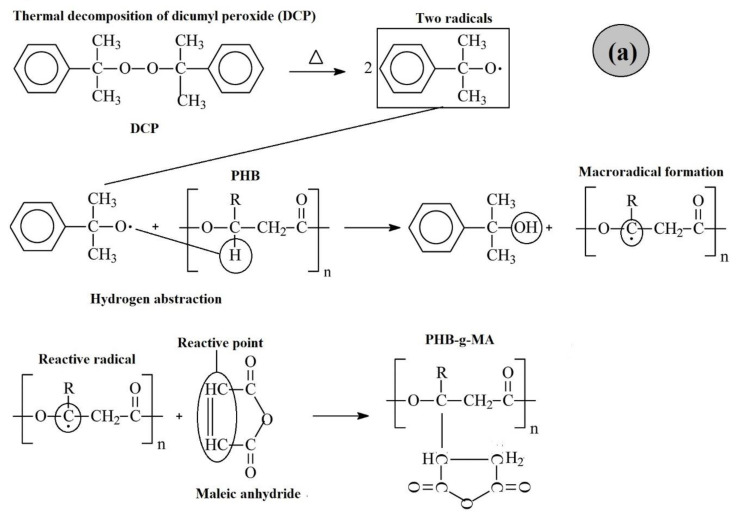

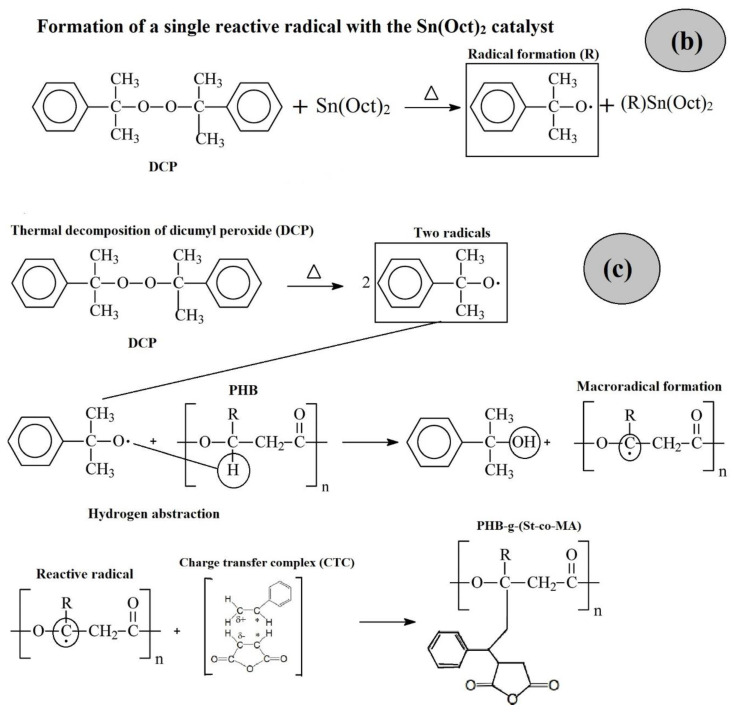
Mechanism of PHB grafting reaction with maleic anhydride, adapted from the literature [61,62]: (**a**) PHB (5 MA/0.5 DCP); (**b**) PHB (5 MA/0.5 DCP/0.5 C); (**c**) PHB (5 MA/0.5 DCP/5 St./0.5 C).

Figure 7 shows PHB maleic anhydride grafting mechanisms using dicumyl peroxide, Sn(Oct)_2_ catalyst, and styrene monomer. The PHB (5 MA/0.5 DCP) system initially occurs with the formation of two primary radicals with the decomposition of DCP. These attack the PHB chain, generating a macroradical with hydrogen abstraction; subsequently, the reactive point reacts with the maleic anhydride. The mechanism for the PHB (5 MA/0.5 DCP/0.5 C) system is similar to the previous one. However, the Sn(Oct)_2_ catalyst attacks a DCP molecule and generates only one free radical (Figure 7b). The styrene monomer increases the reactivity of the maleic anhydride double bonds, generating a charge transfer complex (CTC), which favors a greater grafting degree in the PHB chain. However, the styrene monomer selectively interacts with the PHB macroradial, forming a styrene-stable macroradial (PHB-St•). Subsequently, the reaction to maleic anhydride monomer occurs, generating the multiphase system PHB-g-(St-co-MA) (see Figure 7c).

### 2.4. Contact Angle

**Figure 8:**
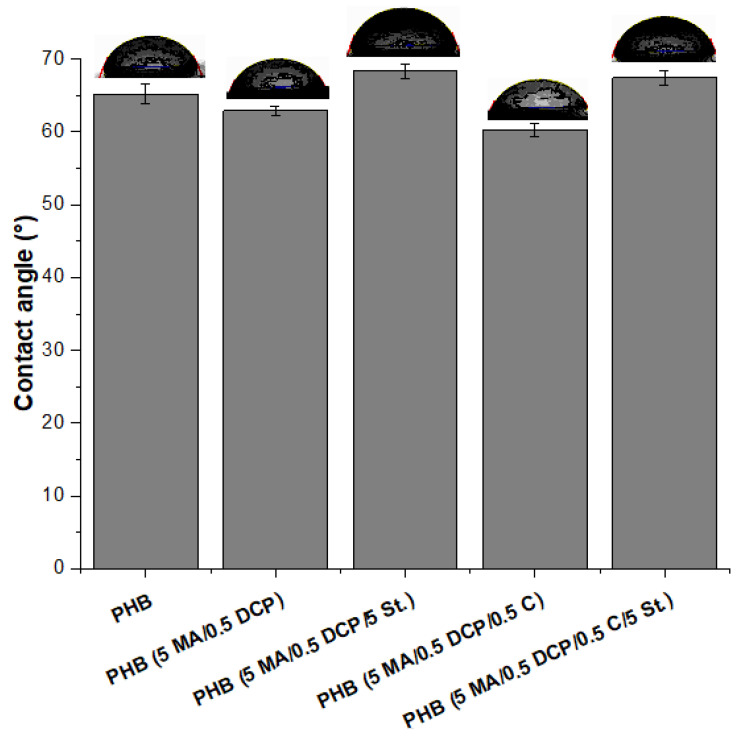
Contact angle of pure PHB and PHB-g-MA.

Figure 8 shows the behavior of the contact angle between PHB and PHB-g-MA, aiming to evaluate how the chemical modification affected the interaction of the surface with water. The contact angle value of pure PHB was around 65.2°, a value close to that reported in the literature [63]. The PHB (5 MA/0.5 DCP) and PHB (5 MA/0.5 DCP/0.5 C) systems slightly reduced the contact angle to 62.9° and 60.3°, respectively. The chemical modification process with maleic anhydride made the surface more hydrophilic, specifically for the PHB (5 MA/0.5 DCP) and PHB (5 MA/0.5 DCP/0.5 C) systems. The increase in polarity was caused by incorporating maleic anhydride into the PHB chain, generating greater affinity with water, and reducing the contact angle, confirming the FTIR spectra. Comparing the PHB (5 MA/0.5 DCP) and PHB (5 MA/0.5 DCP/0.5 C) systems, the most intense band at 1780 cm^−1^ of the FTIR suggested a greater maleic anhydride grafting degree, which reflected in a greater decrease in the contact angle. Given this, the contact angle can be considered an indirect measure to assess the degree of maleic anhydride grafting in the PHB chain. Similar behavior was also verified by Zhao et al. [64] with the process of maleic anhydride grafting onto the polybutene chain. The polybutene contact angle decreased with an increase in the degree of maleic anhydride grafting. The authors suggested that the surface showed greater polarity and hydrophilicity.

Different behavior in the contact angle was verified for the PHB (5 MA/0.5 DCP/5 St.) and PHB (5 MA/0.5 DCP/5 St./0.5 C) systems since they increased to 68.3° and 67.4°, respectively. The incorporation of a styrene monomer made PHB-g-MA more hydrophobic, surpassing the pure PHB contact angle. As the styrene graft in the PHB chain increased, as seen in Figure 6, there was a greater tendency for the contact angle to increase. The styrene monomer is non-polar, forming a barrier effect on the surface of PHB (5 MA/0.5 DCP/5 St.) and PHB (5 MA/0.5 DCP/5 St./0.5 C) samples, minimizing interaction with water and increasing the contact angle. Given this, the PHB-g-(St-co-MA) system is less sensitive to the effect of humidity during thermomechanical processing, generating greater stability against degradation.

### 2.5. X-ray Diffraction (XRD)

**Figure 9:**
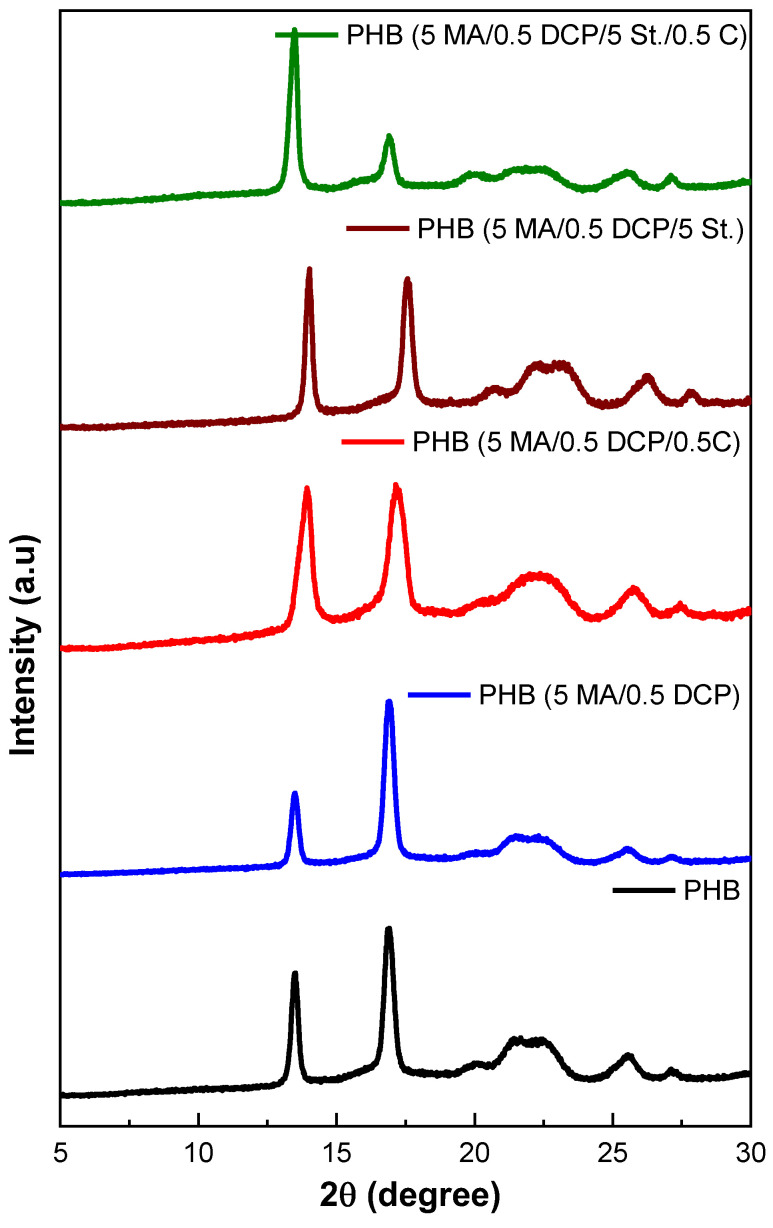
XRD diffractograms for pure PHB and PHB-g-MA.

Figure 9 shows the curves obtained by XRD of PHB and PHB-g-MA as a function of incorporation with styrene (St.), maleic anhydride (MA), DCP, and Sn(Oct)_2_. Data on the degree of crystallinity are summarized in Table 2. The PHB diffractogram illustrates the presence of well-defined crystalline peaks and, at the same time, an amorphous halo, typical of the behavior of semi-crystalline polymers. Two peaks of high intensity were observed around 2θ = 13.49° and 2θ = 16.9°, attributed to planes (020) and (110) [65], respectively. The low-intensity crystalline peaks at 2θ = 19.8°, 2θ = 21.6°, 2θ = 22.1°, 2θ = 25.6°, and 2θ = 27° are from (021), (101), (111), (121), and (040) [66], respectively. The results indicate a crystalline structure with an orthorhombic unit cell, with the parameters a = 5.76 $\dot{A}$, b = 13.2 $\dot{A}\;$e, and c = 5.96 $\dot{A}$ [67]. The peaks at 2θ = 13.49°, 2θ = 21.6°, and 2θ = 22.1° suggest that PHB forms a structure with a predominance of α crystals, as reported in [68]. However, the small peak with low intensity was around 2θ = 19.8° indicates the nucleation of crystals in the β form [69]. This indicates a hybrid population of crystals, α and β, with a predominance of the α form. The literature [70] reported that PHB’s most common crystal structure is in the α-form since it is developed under typical melting conditions.

The XRD spectrum of pure PHB and PHB-g-MA, regardless of the additive used in the chemical modification, shows that the diffraction angles did not change for all crystallographic planes. This indicates that the orthorhombic unit cell of PHB was not altered after the grafting process with maleic anhydride. However, alterations in the intensities of the crystalline peaks were observed with the chemical modification of PHB, indicating a preferential orientation of the crystals in a specific crystalline plane. As reported by Chen et al. [30], the crystalline growth of PHB undergoes variation in different directions. The PHB (5 MA/0.5 DCP) system reduced the diffraction peak intensity at 2θ = 13.49° compared with pure PHB. On the other hand, the PHB (5 MA/0.5 DCP/5 St./0.5 C) suffered a sharp decrease in the diffraction (peak 2θ = 16.9°). This behavior can be explained by a greater restriction of molecular movement after MA grafting, leading to limited growth of crystallites in the (110) plane for the PHB (5 MA/0.5 DCP/5 St./0.5 C) system. Consequently, crystal nucleation is suppressed in the PHB in the (110) plane when styrene monomer and Sn(Oct)_2_ catalyst are used simultaneously, indicating the preferential orientation of crystals within the sample. Samples with a higher maleic anhydride grafting degree prefer crystals in the (020) plane since they increase the intensity of the diffraction (peak 2θ = 13.49°) compared with pure PHB. This finding is confirmed by the diffractograms of the PHB (5 MA/0.5 DCP/0.5 C), PHB (5 MA/0.5 DCP/5 St.), and PHB (5 MA/0.5 DCP/5 St./0.5 C), with the addition of the peak 2θ = 13.49°. Therefore, these changes in intensity values in the crystalline peaks can be attributed to the preferential ordering of the crystalline planes at these diffraction angles.

Table 2 shows the crystallinity degree of pure PHB and PHB-g-MA. Pure PHB showed a high degree of crystallinity with a value of 58.2%, within the crystallinity range of 55–80% for PHB [56]. The maleic anhydride grafting process on the PHB chain caused a decrease in the crystallinity degree of all samples, indicating an inhibition during crystallization. Maleic anhydride grafting onto the PHB chain restricts the organization of the lamellae during crystallization, reducing the formation of crystals, as reported in the literature [21]. The PHB-g-MA (5 MA/0.5 DCP) system presented a degree of crystallinity close to pure PHB, reinforcing the low degree of grafting verified in the titration. When there was an increase in the degree of grafting, there was a tendency for the degree of crystallinity to reduce, as verified in the PHB-g-MA (5 MA/0.5 DCP/0.5 C) sample. PHB-g-MA (5 MA/0.5 DCP/5 St.) and PHB-g-MA (5 MA/0.5 DCP/0.5 C/5 St.) samples showed the lowest degree of crystallinity, due to the higher viscosity, as verified in torque rheometry and complex viscosity. The increase in viscosity causes a loss of molecular mobility, which reduces the probability of forming stable crystals to promote growth.

### 2.6. Differential Scanning Calorimetry (DSC)

**Figure 10:**
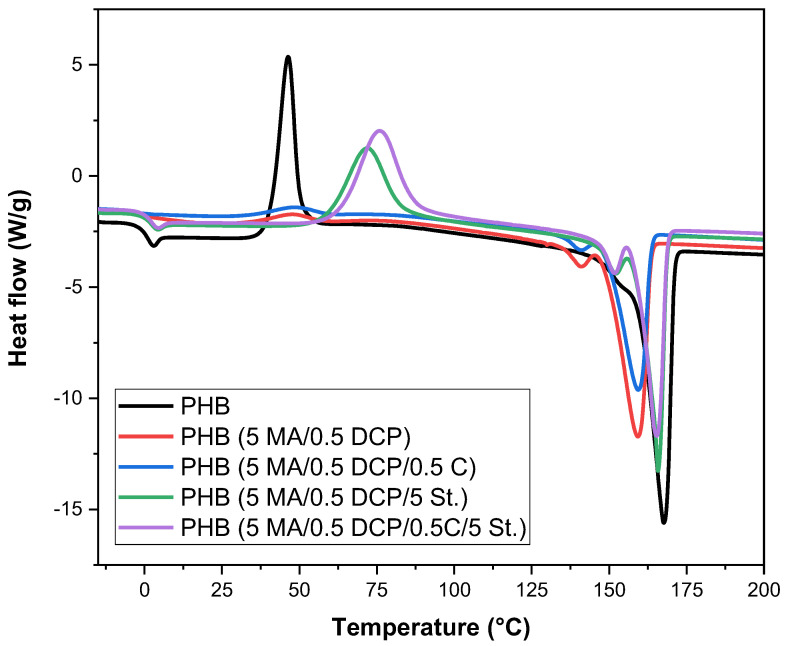
DSC curves for pure PHB and PHB-g-MA during the second heating cycle.

Figure 10 shows the curves obtained by DSC of PHB and PHB-g-MA during the second heating cycle. Table 3 shows the results of the glass transition temperature (T_g_), cold crystallization temperature (T_cc_), and crystalline melting temperature (T_m_). The literature [71] indicates that the T_g_ of PHB is in the range of −5 and 5 ºC. Pure PHB had a T_g_ = 0.7 °C, while the PHB-g-MA (5 MA/0.5 DCP) and PHB-g-MA (5 MA/0.5 DCP/0.5 C) systems shifted to 0.6 °C and −1.1 °C, respectively. As the Tg corresponds to the response of the amorphous fraction of the PHB chain, the PHB-g-MA (5 MA/0.5 DCP) and PHB-g-MA (5 MA/0.5 DCP/0.5 C) systems gained molecular mobility. The opposite effect was verified for PHB-g-MA (5 MA/0.5 DCP/5 St.) and PHB-g-MA (5 MA/0.5 DCP/0.5 C/5 St.) since there was a displacement of the T_g_ for higher temperatures, with values of 1.6 °C and 1.4 °C, respectively. These samples increased the restriction of amorphous chains, generating a loss in molecular mobility. Such behavior can be attributed to the styrene monomer grafting in the PHB chain, as verified by the FTIR. Consequently, the aromatic groups increased resistance to molecular movement, which was reflected in the shift of T_g_ to a higher temperature.

In Figure 10, an exothermic peak was observed in the 40–80 °C range due to the cold crystallization process. This phenomenon is attributed to the rearrangement of amorphous regions in a crystalline phase, as reported in the literature [72]. Pure PHB showed a cold crystallization temperature (T_cc_) of 46.3 °C. The PHB-g-MA (5 MA/0.5 DCP) and PHB-g-MA (5 MA/0.5 DCP/0.5 C) systems maintained the T_cc_ peak, similar to pure PHB. However, a significant reduction in the intensity of the T_cc_ peak was observed, specifically for PHB-g-MA (5 MA/0.5 DCP) and PHB-g-MA (5 MA/0.5 DCP/0.5 C). This indicates that maleic anhydride or dicumyl peroxide additives inhibited the cold crystallization of PHB. On the other hand, the PHB-g-MA (5 MA/0.5 DCP/5 St.) and PHB-g-MA (5 MA/0.5 DCP/0.5 C/5 St.) samples showed a very evident Tcc shift, with values of 75.8 °C and 71.8 °C, respectively. The PHB-g-MA (5 MA/0.5 DCP/5 St.) and PHB-g-MA (5 MA/0.5 DCP/0.5 C/5 St.) systems had accelerated nucleation at T_cc_. Incorporating a styrene monomer was important to accelerate the cold crystallization process, acting as a nucleating agent for this phenomenon in the PHB matrix.

The PHB crystalline melting temperature (T_m_) showed an intense peak around 167.6 °C, attributed to the melting of the most thermally stable crystalline form, the α phase. In comparative terms, pure PHB showed a higher perfection degree of the crystals among all the materials in Figure 10, which requires more energy to melt. By submitting PHB to the maleic anhydride grafting process, the crystalline melting temperature tends to reduce. As the reduction in the crystalline melting temperature is associated with the perfection degree of the crystals [73], it is suggested that the maleic anhydride grafting process in the PHB chain affected the thickness of the lamellae, reducing their degree of perfection. The PHB-g-MA (5 MA/0.5 DCP) and PHB-g-MA (5 MA/0.5 DCP/0.5 C) systems showed the most severe reductions in the crystalline melting temperature, suggesting a greater imperfection degree in the crystals. When styrene monomer was incorporated into the chemical modification, PHB-g-MA (5 MA/0.5 DCP/5 St.) and PHB-g-MA (5 MA/0.5 DCP/0.5 C/5 St.), there was a tendency to shift T_m_ to a higher temperature. Such behavior indicates that the styrene monomer improved the maleic anhydride grafting degree and, at the same time, maintained the formation of more thermally stable crystals. Given this, it is suggested that the PHB-g-MA (5 MA/0.5 DCP/5 St.) and PHB-g-MA (5 MA/0.5 DCP/0.5 C/5 St.) systems nucleate crystals with lamellae thicker, increasing the perfection degree and the Tm. When PHB was functionalized with maleic anhydride, a small melting peak was formed in the range of 130–155 °C. This finding suggests a heterogeneity in the size of the crystals, confirming the tendency of the XRD. Furthermore, the addition of styrene monomer tended to shift the small melting peak to a higher temperature, as seen in PHB-g-MA (5 MA/0.5 DCP/5 St.) and PHB-g-MA (5 MA/0.5 DCP/0.5 C/5 St.). 

### 2.7. Thermogravimetry (TG)

**Figure 11:**
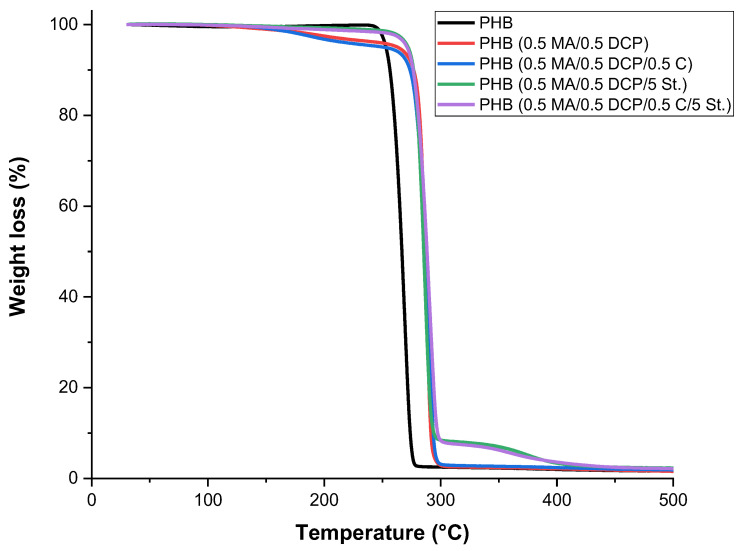
Thermogravimetry for pure PHB before and after chemical modification.

Figure 11 illustrates the behavior of the thermal stability of pure PHB and PHB-g-MA as a function of the additives. It was observed that pure PHB suffered severe thermal degradation above 250 °C, with the material being wholly decomposed in a single step up to 300 °C without generating waste. The literature [40,74] indicates that the thermal degradation mechanism of PHB is based on the random scission of ester groups, generating the elimination of a β hydrogen. The PHB (5 MA/0.5 DCP) and PHB (5 MA/0.5 DCP/0.5 C) samples showed a slight decrease in the TG curve in the 170–260 °C range, referring to the loss of maleic anhydride. By adding styrene monomer, the PHB (5 MA/0.5 DCP/5 St) and PHB (5 MA/0.5 DCP/5 St./0.5 C) systems maintained thermal stability at a constant level; that is, there was no decrease in the curve at a temperature below 300 °C. This suggests that styrene promoted a barrier effect protecting the maleic anhydride functional group, generating greater resistance to thermal decomposition. The most important finding was that the thermal decomposition temperature of the PHB chain increased after MA grafting for all samples. The expansion of the thermal stability of PHB after the maleic anhydride graft is of great importance for the industrial sector, considering the processing in extruder and injection.

Table 4 shows the temperature for 10% and 50% mass loss of pure PHB before and after chemical modification. PHB samples with maleic anhydride grafting increased on average 20 °C stability for 10% and 50% weight loss compared with pure PHB. In comparative terms, the PHB-g-MA samples showed similar T_0.1_ and T_0.5_ parameters in mass loss, with no significant differences. Consequently, it is suggested that the thermal stability of PHB-g-MA is associated with the degree of maleic anhydride grafting and the grafting location. Substitution of unstable protons on tertiary carbons of PHB may increase thermal stability, generating steric hindrance. The literature [45,46] suggests that a strong steric hindrance with the incorporation of maleic anhydride would block the formation of the six-member ring in the PHB chain, directly reflecting an improvement in the thermal stability of the PHB.

## 3. Materials and Methods

### 3.1. Materials

Polyhydroxybutyrate (PHB), marketed under the code FE-113, with a melt flow index (MFI) of 30 g/10 min (2.16 kg/190 °C—ASTM D-1238) and M_w_ = 340 kg/mol, is produced by PHB Industrial SA (Brazil). Maleic anhydride (MA) with 95% purity was used as a functional group in the form of flakes, manufactured by Sigma Aldrich. Dicumyl peroxide (DCP) was used as a free radical initiator in powder form with 98% purity and manufactured by Sigma Aldrich. Styrene (St) was used as a comonomer with 99% purity and was manufactured by Sigma Aldrich. Tin(II) 2-ethylhexanoate (Sn(Oct)_2_) was used as a catalyst with 99% purity and manufactured by Sigma Aldrich.

### 3.2. Chemical Modification of PHB

Before processing, the PHB was dried in a vacuum oven for 24 h at a temperature of 60 °C. The chemical modification of PHB with maleic anhydride was carried out in a torque rheometer, Thermocientific Polylab QC, with roller-type rotors at a temperature of 180 °C and a rotor speed of 60 rpm under an air atmosphere for 10 min. Table 5 shows the proportions of maleic anhydride (MA), dicumyl peroxide (DCP), styrene monomer (St.), and Sn(Oct)_2_ catalysts used during PHB functionalization. The maleic anhydride/styrene ratio adopted was 1:1, as suggested in the literature [31,41]. The additives (MA, DCP, and Sn(Oct)_2_) were added to the PHB after 2 min of processing when the PHB was already molten. The polyhydroxybutyrate grafted with maleic anhydride (PHB-g-MA) obtained was ground in a knife mill and subsequently purified to be characterized.

The PHB-g-MA was dried in a vacuum oven at 60 °C and purified to remove unreacted maleic anhydride. A 20 g sample of PHB-g-MA was dissolved in 300 mL of boiling chloroform and refluxed for 2 h. The dissolved solution was precipitated with methanol. The precipitated PHB-g-MA was vacuum filtered and washed with methanol to remove ungrafted MA from the PHB.

### 3.3. Material Characterizations

The rheological curves were obtained in a Thermo Scientific Haake PolyLab QC mixer with roller-type rotors at a temperature of 180 °C and a rotor speed of 60 rpm under an air atmosphere, for 10 min. The viscosity (η) was determined through Equation (1) [75], using the dimensions of the torque rheometer, the rotation speed of the rotors, and the torque value.

(1) $$\eta =\frac{T}{N}\frac{\left(\beta^{2}-1\right)}{8\pi^{2}LR_{e}^{2}(1+g^{2})}\;\;$$

where T is the torque; β = R_e_/R_i_, where R_e_ is the radius of the chamber and R_i_ is the radius of the rotors; N is the speed of the rotors; g = N_2_/N_1_ is the ratio between the secondary (N_2_) and primary (N_1_) speeds of the rotors; L is the length of the rotors. The torque rheometer parameters used are reported in the literature [74]. The torque values were obtained in 10 min of the experiment, and the speed of the rotors was 60 rpm (1 s^−1^).

The rheological behavior of PHB was evaluated in a dynamic state under the nitrogen atmosphere (AR-G2 stress-controlled rheometer, TA Instruments, New Castle, NY, USA) and parallel-plate geometry with 25 mm of diameter and a gap between plates of 1 mm. The operational conditions were at a temperature of 180 °C. The deformation adopted was 1% within the linear viscoelasticity region. The analysis was performed on rectangular samples taken from impact specimens.

Fourier transform infrared spectroscopy (FTIR) was performed on a BRUKER Vertex 70 Spectrometer (total attenuated reflectance) in the range of 4000 to 400 cm^−1^, with 64 scans and a resolution of 4 cm^−1^.

The degree of maleic anhydride grafting was determined by a titration procedure on purified samples of PHB-g-MA using a route similar to that described in the literature [76,77,78]. A 1 g sample was refluxed for 1 h in 100 mL of boiling chloroform. The dissolved solution was titrated with 0.1 N ethanolic potassium hydroxide (KOH), using 1% thymol blue drops in dimethylformamide as an indicator. A blank test (pure PHB) was performed with the same method, and consequently, the grafted MA content (% g-MA) was reported according to Equation (2):

(2) $$\%g-\mathrm{MA}=\frac{N\left(V_{2}-V_{1}\right)\times \;98.06}{2\times W\times 1000}\times 100\%$$

where N = KOH normality; *V*_1_ = pure PHB titration volume; *V*_2_ = PHB-g-MA titration volume; *W* = sample mass. The grafting efficiency (E_f_) of maleic anhydride in PHB was determined using Equation (3) [79]:

(3) $$E_{f}=\frac{m_{1}}{m_{0}}\times 100\%$$

where *m*_1_ = weight of grafted MA calculated through the grafting degree; *m*_0_ = MA weight initially added.

The contact angle was determined through the sessile drop method using portable contact angle equipment, model Phoenix-i by Surface Eletro Optics—SEO. The drop was deposited on the sample in the form of a film using a micrometric doser. The captured image was analyzed by the software associated with the equipment in 200 s of stability.

Differential scanning calorimetry (DSC) analysis was performed on a Mettler Toledo DSC-1 apparatus. The test was conducted from −30 to 200 °C under a heating rate of 10 °C/min, a gas flow rate of 50 mL/min, a nitrogen atmosphere, and using 6 mg of sample. 

Thermogravimetry (TG) analysis was obtained in Shimadzu DTG-60H equipment using about 7 mg of sample, with a heating rate of 10 °C/min and a gas flow of 50 mL/min, starting from room temperature (~30 °C) to 500 °C under a nitrogen atmosphere.

X-ray diffraction analysis (XRD) was carried out in a Bruker D2 Phaser diffractometer, using copper Kα radiation, a voltage of 30 kV, a current of 10 mA, a 2θ sweep from 5° to 30°, and a speed of 2°/min. 

## 4. Conclusions

Polyhydroxybutyrate (PHB) was functionalized in the molten state with maleic anhydride and dicumyl peroxide, using styrene monomer and Sn(Oct)_2_ catalyst to enhance the grafting degree. Functionalization by the direct method of PHB with MA/DCP showed low efficiency, with a graft of 0.23%. The incorporation of the Sn(Oct)_2_ additive in a small amount (0.5 phr) increased the grafting degree to 0.39%, suggesting greater stabilization in the reaction medium. The styrene monomer increased the maleic anhydride reactivity, improving the grafting degree performance. However, the simultaneous combination of Sn(Oct)_2_/St (0.5/5 phr) did not promote improvement in the grafting degree of PHB, only a negative effect. Torque rheometry and complex viscosity indicated chain scission for PHB (5 MA/0.5 DCP) and PHB (5 MA/0.5 DCP/0.5 Sn(Oct)_2_). On the other hand, the increase in viscosity with the addition of styrene monomer indicated simultaneous grafting between maleic anhydride and styrene. This finding was corroborated by the FTIR analysis, considering the emergence of bands at 1780 cm^−1^ and 704 cm^−1^, generating the multiphasic component PHB-g-(St-co-MA). The reduction in the crystallinity degree, the glass transition temperature, and the crystalline melting temperature indicated that maleic anhydride grafting in the PHB chain affected crystal perfection and molecular mobility. An interesting finding was the shift in thermal stability at 20 °C with the grafting process on the PHB chain. This makes it important for the industrial sector, given the increased thermal stability for processing in an extruder or injection. 

The results presented for the maleic anhydride grafting onto the PHB chain are essential for polymer synthesis through free radicals and in the molten state. Firstly, the simultaneous combination of the styrene monomer and the Sn(Oct)_2_ catalyst did not favor the degree of MA grafting onto the PHB chain. In this case, the cost of the final product is minimized due to reduced additive consumption. Secondly, synthesizing PHB modified with MA can favor consuming a material with sustainable characteristics. The chemical modification of PHB with maleic anhydride is significant in expanding the range of applications as a reactive compatibilizer for producing polymeric blends, composites, and nanocomposites.

## Figures and Tables

**Table 1:** Stabilized average torque in the 8–10 min interval and the viscosity (η) obtained through torque rheometry in 10 min.

Samples *	Average Torque (N.m)	η (Pa.s) **
PHB	2.1 ± 0.04	41.7
PHB-g-MA (5 MA/0.5 DCP)	1.2 ± 0.02	22.3
PHB-g-MA (5 MA/0.5 DCP/5 St.)	2.6 ±0.03	52.9
PHB-g-MA (5 MA/0.5 DCP/0.5 C)	1.3 ± 0.01	25.1
PHB-g-MA (5 MA/0.5 DCP/0.5 C/5 St.)	2.3 ± 0.04	47.3

* MA = maleic anhydride; DCP = dicumyl peroxide; St. = styrene monomer; C = Sn(Oct)_2_ catalyst. ** Adopted the terminal torque in 10 min of process.

**Table 2:** Degree of crystallinity of PHB and PHB-g-MA obtained by XRD.

Samples	Crystallinity (%)
PHB	58.2
PHB-g-MA (5 MA/0.5 DCP)	55.8
PHB-g-MA (5 MA/0.5 DCP/5 St.)	51.6
PHB-g-MA (5 MA/0.5 DCP/0.5 C)	53.9
PHB-g-MA (5 MA/0.5 DCP/0.5 C/5 St.)	48.5

**Table 3:** Parameters obtained during heating of PHB and PHB-g-MA.

Samples	T_g_ (°C)	T_cc_ (°C)	T_m_ (°C)
PHB	0.7	46.3	167.6
PHB-g-MA (5 MA/0.5 DCP)	0.6	47.6	159.3
PHB-g-MA (5 MA/0.5 DCP/5 St.)	1.6	71.8	165.9
PHB-g-MA (5 MA/0.5 DCP/0.5 C)	−1.1	48.2	159.7
PHB-g-MA (5 MA/0.5 DCP/0.5 C/5 St.)	1.4	75.8	165.3

**Table 4:** Thermal decomposition parameters of PHB and PHB-g-MA for 10% (T_0.1_) and 50% (T_0.5_) mass loss.

Samples	T_0.1_ (°C)	T_0.5_ (°C)
PHB	256.3	266.7
PHB-g-MA (5 MA/0.5 DCP)	276.7	286.6
PHB-g-MA (5 MA/0.5 DCP/5 St.)	276.7	285.5
PHB-g-MA (5 MA/0.5 DCP/0.5 C)	273.6	286.7
PHB-g-MA (5 MA/0.5 DCP/0.5 C/5 St.)	276.3	288.8

**Table 5:** Proportions of the additives used to functionalize the PHB.

Sample	PHB (% Weight)	MA (phr)	DCP (phr)	St. (phr)	Sn(Oct)_2_ (phr)
PHB	100	-	-	-	-
PHB (MA/DCP)	100	5	0.5	-	-
PHB (MA/DCP/St.)	100	5	0.5	5	-
PHB (MA/DCP/C)	100	5	0.5	-	0.5
PHB (MA/DCP/St./C)	100	5	0.5	5	0.5

phr = parts per hundred of resin; Dicumyl peroxide = DCP; Styrene monomer = St.; C = Sn(Oct)_2._

## Data Availability

Not applicable.
